# Androgen receptor-dependent and -independent mechanisms driving prostate cancer progression: Opportunities for therapeutic targeting from multiple angles

**DOI:** 10.18632/oncotarget.12554

**Published:** 2016-10-10

**Authors:** David T Hoang, Kenneth A Iczkowski, Deepak Kilari, William See, Marja T Nevalainen

**Affiliations:** ^1^ Sidney Kimmel Medical College, Thomas Jefferson University, Philadelphia, PA, USA; ^2^ Department of Pathology, Medical College of Wisconsin Cancer Center, Medical College of Wisconsin, Milwaukee, WI, USA; ^3^ Department of Medicine, Medical College of Wisconsin Cancer Center, Medical College of Wisconsin, Milwaukee, WI, USA; ^4^ Department of Urology, Medical College of Wisconsin Cancer Center, Medical College of Wisconsin, Milwaukee, WI, USA; ^5^ Department of Pharmacology/Toxicology, Medical College of Wisconsin Cancer Center, Medical College of Wisconsin, Milwaukee, WI, USA

**Keywords:** androgen receptor, castrate-resistant, antiandrogen, metastasis, Jak2, Stat5a/b, prostate cancer

## Abstract

Despite aggressive treatment for localized cancer, prostate cancer (PC) remains a leading cause of cancer-related death for American men due to a subset of patients progressing to lethal and incurable metastatic castrate-resistant prostate cancer (CRPC). Organ-confined PC is treated by surgery or radiation with or without androgen deprivation therapy (ADT), while options for locally advanced and disseminated PC include radiation combined with ADT, or systemic treatments including chemotherapy. Progression to CRPC results from failure of ADT, which targets the androgen receptor (AR) signaling axis and inhibits AR-driven proliferation and survival pathways. The exact mechanisms underlying the transition from androgen-dependent PC to CRPC remain incompletely understood. Reactivation of AR has been shown to occur in CRPC despite depletion of circulating androgens by ADT. At the same time, the presence of AR-negative cell populations in CRPC has also been identified. While AR signaling has been proposed as the primary driver of CRPC, AR-independent signaling pathways may represent additional mechanisms underlying CRPC progression. Identification of new therapeutic strategies to target both AR-positive and AR-negative PC cell populations and, thereby, AR-driven as well as non-AR-driven PC cell growth and survival mechanisms would provide a two-pronged approach to eliminate CRPC cells with potential for synthetic lethality. In this review, we provide an overview of AR-dependent and AR-independent molecular mechanisms which drive CRPC, with special emphasis on the role of the Jak2-Stat5a/b signaling pathway in promoting castrate-resistant growth of PC through both AR-dependent and AR-independent mechanisms.

## INTRODUCTION

Recent epidemiological data identifies prostate cancer (PC) as the most common non-cutaneous cancer and the second-leading cause of cancer-related death among males in the United States following lung cancer [1]. According to the American Cancer Society, approximately 180,000 new cases of PC are diagnosed and 26,000 men, or 1 in 39, die of PC each year [1]. The clinical course of PC is heterogeneous, ranging from indolent to rapidly progressive and fatal. While the five-year survival rate for localized PC is close to 100% due to the availability of curative treatments, some patients experience cancer progression to metastatic castrate-resistant prostate cancer (CRPC), which is currently incurable and carries a poor prognosis (reviewed in [2–4]). Although the recent U.S. Food and Drug Administration (FDA) approval of numerous therapeutic agents for CRPC is promising, an unmet need still exists for the development of rational biomarkers and novel treatment strategies to improve survival.

Prior to 2010, the chemotherapeutic taxane docetaxel (Taxotere®) was the only drug demonstrated to improve survival of CRPC patients in comparison to palliative chemotherapy with mitoxantrone (Novantrone®), increasing median overall survival from 16.3 to 19.2 months [5, 6]. In the last several years, there has been an influx of new therapies mainly due to improved understanding of CRPC biology [4, 7]. These promising drugs have positively altered the therapeutic landscape of CRPC, but emerging resistance mechanisms have already been described for most of these agents (reviewed in [8–11]).

The therapeutic agents receiving FDA approval for treatment of advanced PC in the past five years include 1) abiraterone (Zytiga®; approved 2011), 2) enzalutamide (Xtandi®; approved 2012), 3) cabazitaxel (Jevtana®; approved 2010), 4) sipuleucel-T (Provenge®; approved 2010) and 5) Alpharadin (Xofigo®; approved 2013) (reviewed in [4, 12–14]). Abiraterone is a small-molecule inhibitor of cytochrome P450 17A1 (CYP17A1), an enzyme required for both adrenal and intratumoral de novo biosynthesis of androgens [15]. Enzalutamide is a second-generation antiandrogen and acts as a pure antagonist with no agonist activity [16, 17]. Cabazitaxel is a third-generation chemotherapeutic of the taxane class, which demonstrated superiority to palliative mitoxantrone-based chemotherapy in the post-docetaxel metastatic CRPC setting, although the use of the drug has been hampered by hematological adverse events, most notably febrile neutropenia [18]. Sipuleucel-T is an autologous cellular immunotherapy, also referred to as a therapeutic cancer vaccine, designed to generate an immune response against PC cells expressing prostatic acid phosphatase [19, 20]. Alpharadin is a radioisotope-containing radium-223 dichloride, a nuclide which emits alpha particles, that allows for targeting of PC bone metastases with short-range, high-energy alpha radiation [21]. Clinical trials that investigate the optimal sequence [7, 14] and combinations of these agents in advanced PC to minimize side effects and exploit synergistic mechanisms are needed. Most importantly, novel agents which can be deployed to impose synthetic lethality [22, 23] or applied as second- or third-line treatments [24] in the setting of resistance to current therapies need to be identified and further developed. The clinical limitations of a narrow focus on androgen receptor (AR) as the sole therapeutic target in PC have been increasingly recognized as resistance to any agents targeting AR is inevitable [25–27]. Investigational approaches using combination therapy with pharmacological agents directed against AR and other molecular targets, in addition to AR-negative cells, in advanced PC may prove to be critical to enhance efficacy and delay onset of resistance to agents targeting AR in PC.

## THERAPEUTIC TARGETING OF AR IN PROSTATE CANCER GROWTH

Similar to normal prostate, PC cells require androgens for continued growth [28]. The requirement for androgens is effectively exploited through the use of ADT as a first-line therapy for advanced PC (reviewed in [29–31]). AR, a member of the steroid hormone group of nuclear receptors which includes estrogen receptor (ER), progesterone receptor (PR) and mineralocorticoid receptor (MR), functions as a ligand-dependent transcription factor. Binding of androgen ligands, such as testosterone and the more potent dihydrotestosterone (DHT), induces a conformational change in AR that allows for nuclear translocation and induction of androgen-responsive gene expression supporting growth and viability of prostate cells (reviewed in [32–36]). Mechanistically, AR inhibition induces both cell cycle arrest and apoptosis of PC cells [37–41]. Expression of AR is found in primary PC and continues to be detectable throughout progression to CRPC [42, 43].

ADT by means of surgical or pharmacological castration, the latter in the form of luteinizing hormone-releasing hormone (LHRH) / Gonadotropin-releasing hormone (GnRH) agonists (e.g. leuprorelin (Lupron®); goserelin (Zoladex®)) reduces serum testosterone levels by 90-95%. This robust decrease in serum androgens is dampened by the fact that intraprostatic levels of DHT have been reported to decline by only about 50% [44]. The stimulus from the residual intraprostatic DHT and other androgens can be blocked by addition of an antiandrogen drug to produce combined androgen blockade (CAB) [4, 12, 45, 46]. First-generation antiandrogens such as flutamide and bicalutamide have been used clinically for decades and function as AR antagonists by competitive inhibition of androgen binding to AR [4, 12, 45, 46]. In the case of bicalutamide, it also promotes recruitment of AR corepressors which contributes to inhibition of AR transcriptional activity [47]. The second-generation antiandrogen enzalutamide, designed to bind the AR ligand-binding domain (LBD), induces a conformational change in AR that prevents AR nuclear translocation to a greater extent than first-generation agents [16, 17]. In contrast to the use of LHRH/GnRH agonists alone, the addition of antiandrogens should theoretically prevent residual intraprostatic DHT from binding to AR. However, meta-analyses of clinical trials comparing CAB vs. LHRH/GnRH agonist monotherapy for advanced PC have demonstrated only a modest benefit in overall survival, often outweighed by added toxicity and decreased quality of life [48–50].

## PROGRESSION OF PROSTATE CANCER TO CASTRATE-RESISTANT DISEASE

PC is considered castrate resistant during ADT with castrate levels of serum testosterone (<50 ng/dL) if it fullfils one or more of the following criteria: 1) a rise of prostate-specific antigen (PSA) serum levels (biochemical progression), 2) development of symptoms in the presence of pre-existing cancer (clinical progression) or 3) detection of new metastatic lesions on imaging (radiographic progression) [51, 52]. CRPC has historically been referred to by several names, including “hormone-refractory” and “androgen-independent” PC [53]; however, the preferred terminology is “castrate-resistant” in recognition of intracrine androgen production which is at least partially responsible for resistance to ADT [54, 55]. CRPC presents as a spectrum of disease, ranging from asymptomatic rising PSA levels without evidence of metastasis to multiple distant metastases with debilitating cancer-related constitutional symptoms.

**Figure 1:**
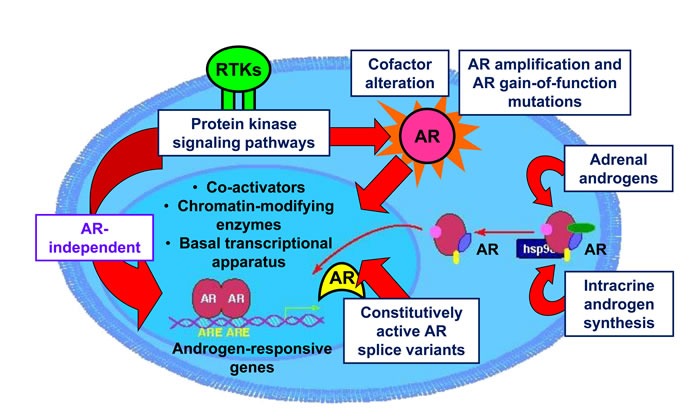
Molecular mechanisms driving CRPC Development of CRPC has been attributed to numerous potential molecular mechanisms, including: 1) somatic mutations of AR resulting in increased affinity for ligands; 2) amplification of the AR gene locus; 3) intracrine biosynthesis of androgens in prostate cancer cells from adrenal steroids and cholesterol; 4) expression of constitutively active, ligand-independent AR splice variants; 5) non-canonical activation of AR by protein kinase signaling pathways in the absence of ligand by receptor tyrosine kinases (RTK); 6) AR-independent mechanisms operating outside of the AR signaling axis which promote castrate-resistant growth of prostate cancer.

The mechanisms driving progression from androgen-dependent PC to CRPC are still largely unclear. Continued AR signaling, despite depletion of circulating androgens and AR blockade, is thought to be central to the development of CRPC [56, 57]. Reactivation of AR transcriptional activity has been attributed to various mechanisms, yet no single mechanism has been reliably shown to account completely for progression to CRPC in experimental models or clinical patients. The majority of research efforts have sought to understand how AR signaling is restored in CRPC, but a growing number of studies highlight mechanisms operating outside the AR signaling axis, which may substantially contribute to the development of CRPC. Figure 1 provides a summary of the most widely investigated mechanisms thought to drive CRPC.

## AR-DEPENDENT MOLECULAR MECHANISMS DRIVING CASTRATE-RESISTANT PROSTATE CANCER

### AR gene amplification and overexpression

One mechanism of AR reactivation in CRPC is via increased expression of AR, which may be achieved through genomic amplification of the AR locus or upregulation of AR protein levels. Early work using patient-matched clinical specimens indicated that approximately 30% of CRPC tumors harbored high-level AR gene amplification as demonstrated by fluorescence in situ hybridization (FISH), in comparison to none of the androgen-dependent primary tumors [58, 59]. Increased AR expression can sensitize PC cells to sub-physiological levels of androgen [60] and overcome inhibition by antiandrogens such as bicalutamide [61]. PC cells which overexpress AR are capable of accessing a greater number of binding sites on chromatin, generating an altered AR transcriptome [62]. In one report, cDNA microarray analysis of CRPC xenograft tumors found that the only change consistently associated with antiandrogen resistance was a modest increase in AR mRNA levels [63].

### Somatic AR mutations

Somatic AR mutations may occur selectively in response to androgen deprivation, although the exact frequency of AR mutations in PC remains unknown. A review of 27 clinical studies found that AR mutations in androgen-dependent tumors ranged from 2-25%, while the incidence in CRPC tumors was slightly higher at 10-40% [64]. Additional work has identified the AR LBD as a mutational hotspot, with more recent studies placing the incidence of AR LBD point mutations in CRPC at ~15-20% [65–68]. Several gain-of-function AR LBD mutations which confer hypersensitivity to androgens or broaden ligand specificity have been characterized. Two well-known examples are AR-T877A and AR-W741C, originally described in LNCaP cells, which convert the antiandrogens flutamide and bicalutamide to partial agonists, respectively [69, 70].

Resistance to the second-generation antiandrogens enzalutamide (formerly MDV3100) and apalutamide (formerly ARN-509) was shown to be conferred by the novel AR LBD mutation F876L (alternatively reported as F877L in a different genomic build) in in vitro and in vivo models of PC [25–27]. Moreover, AR-F876L was also detected circulating as cell-free tumor DNA in the serum of patients who had received apalutamide as part of a phase I clinical trial [26]. AR-F876L repositions the co-activator docking helix 12 of AR, promoting an antagonist-to-agonist switch of enzalutamide and apalutamide [25–27]. However, the overall contribution of AR-F876L to clinical resistance to second-generation antiandrogens remains to be determined; a genomic landscape study of 150 men with metastatic CRPC using next-generation sequencing did not detect the mutation, despite some patients previously receiving enzalutamide [68].

Analysis of plasma from patients with metastatic CRPC for circulating cell-free tumor DNA detected several AR LBD mutations associated with abiraterone resistance, including AR-T878A and AR-L702H [71, 72]. In one such study, emergence of AR-T878A and AR-L702H was observed in 13% of CRPC tumors progressing on abiraterone, while total AR copy number remained unchanged pre- and post-treatment with abiraterone [71]. Mechanistically, these AR LBD mutants were demonstrated to sensitize AR to activation by progesterone (T878A) [73] and glucocorticoids (L702H) [74], circumventing the effects of CYP17A1 inhibition on AR signaling. Interestingly, it is necessary for abiraterone to be administered concurrently with the glucocorticoid prednisone to prevent the common adverse reaction of mineralocorticoid excess syndrome resulting from prolonged CYP17A1 blockade [74].

### Prostate intracrine androgen biosynthesis

In the absence of circulating androgens produced by the testes, an alternative mechanism to activate AR signaling is through conversion of cholesterol and adrenal androgen precursors into testosterone and DHT in PC cells (reviewed in [75, 76]). It has been demonstrated that PC cells contain all components required for androgen biosynthesis, with many of the key enzymes being elevated in recurrent or metastatic tumors [77, 78]. Enzymes responsible for the conversion of cholesterol to androgen precursors (CYP17A1, HSDD3B2) and conversion of androgen precursors to testosterone and DHT (AKR1C3, SRD5A1/2) were shown to be elevated approximately 8-10 fold in metastatic PC [79]. Therefore, upregulation of the androgen production machinery may increase local androgen concentrations in CRPC, further facilitating reactivation of the AR signaling pathway. Production of androgen precursors, such as dehydroepiandrosterone (DHEA) and androstenedione, is blocked by abiraterone, which inhibits the enzymatic activity of the CYP17A1 enzyme [80, 81]. Other androgen biosynthesis inhibitors in clinical development are Ortonel (TAK-700), Galeterone (TOK-001/VN1/124-1) and VT-464 [46]. However, several mechanisms of resistance to abiraterone were documented shortly after FDA approval, including elevated intratumoral levels of CYP17A1 [82] and gain-of-function mutations which sensitize AR to non-androgen ligands [73, 74]; it remains to be seen whether these represent the primary means by which resistance develops to abiraterone.

### AR splice variants (AR-Vs)

Constitutively active AR splice variants (AR-Vs), generated through alternative gene splicing or rearrangement of the AR gene, have been increasingly studied as a contributor to castrate-resistant growth over the past several years. The expression of AR-Vs has now been well-documented in PC cell lines, xenograft tumors, and clinical PC samples [83–85]. The majority of AR-Vs have extensive truncation or exon skipping of the complete C-terminus, leading to loss of the AR LBD. AR-Vs, in which the bipartite nuclear localization signal (NLS) contained in exons 3 and 4 is disrupted, were shown to be predominantly cytoplasmic in AR transactivation reporter assays [86]. However, other AR-Vs contain both exons 3 and 4 and are thus capable of nuclear translocation and activation of AR target genes in a ligand-independent manner. A notable exception is AR-V7, the best characterized AR-V to date, which is constitutively active despite lacking a full NLS, through a mechanism yet to be fully determined [84, 86, 87].

Whether AR-Vs promote castrate-resistant growth of PC primarily by reactivating the normal AR transcriptome or generating an altered AR gene signature remains disputed. Although AR-Vs have been shown to be capable of activating canonical AR target genes such as KLK3 (encoding PSA), several studies have investigated whether AR-Vs can generate alternative gene signatures in CRPC cells [85–87]. One report found that the AR-V gene signature was largely independent of the transcriptional profile generated by full-length AR [88]. In contrast, the Dehm group found that AR-Vs, including AR-V7, preferentially bind the same canonical high-affinity androgen response elements (AREs) engaged by full-length AR on a genome-wide scale [89]. Of note, the Dehm group developed a unique model, the R1-D567 cell line, engineered to exclusively express constitutively active AR-Vs in the absence of full-length AR [89], while other investigators have used PC cell lines with heterogeneous expression of both full-length AR and AR-Vs [85–87]. These findings suggest that AR-V genomic binding specificity and transcriptional output may be highly dependent on the experimental model selected and its particular cellular context.

The constitutive, ligand-independent activity of AR-Vs has substantial implications for treatment of CRPC. AR-V7 transcript and protein levels were found to be upregulated in clinical CRPC bone metastases and correlated with decreased survival rate after surgery [90]. In a more recent study, detection of AR-V7 in circulating tumor cells from enzalutamide- or abiraterone-treated patients was shown to be associated with therapeutic resistance to both agents [91]. AR-V7-positive patients treated with either enzalutamide or abiraterone were found to have lower PSA response rates, shorter PSA progression-free survival, shorter clinical or radiographic progression-free survival and shorter overall survival, compared to AR-V7-negative patients [91]. Based on the noted association, disruption of AR-V activity may be a means to prevent or reverse resistance to enzalutamide and abiraterone. Indeed, short-interfering RNA (siRNA)-mediated knockdown of AR-V7 was shown to confer sensitivity to enzalutamide in CWR22Rv1 cells, which intrinsically express high levels of AR-V7 [92]. On the other hand, overexpression of AR-V7 in LNCaP cells negative for AR-Vs failed to confer resistance to enzalutamide in vitro or in vivo [86]. Further mechanistic work is therefore needed to determine the contribution of AR-V7 and other AR-Vs to development of resistance to enzalutamide and abiraterone.

A novel therapeutic strategy capable of inhibiting both full-length AR and AR-Vs relies on targeting of the AR N-terminus, which is conserved across all AR isoforms. EPI-001, a bisphenol A-derived small molecule antagonist, which covalently binds the AR N-terminal domain, was shown to block transcriptional activity of AR and several AR-Vs, including AR-V7 [93–96]. Based on the preclinical data, a prodrug formulation of the compound, EPI-506, is currently undergoing a Phase I/II clinical trial (NCT02606123) in CRPC patients previously treated with enzalutamide and/or abiraterone. Targeting of the AR N-terminus may prove to be a viable therapeutic approach for circumventing AR LBD-based deletions, which confer resistance to conventional antiandrogens and ligand-depleting agents such as abiraterone.

### Non-canonical AR transactivation

Numerous growth factors, cytokines, and hormones have been implicated in the activation of AR when androgens are absent or present at sub-physiological concentrations [97]. Non-canonical induction of AR signaling has been linked to mechanisms which promote AR phosphorylation [98]. Insulin-like growth factor-1 (IGF-1) was shown to induce AR transcriptional activity under androgen-deprived conditions in vitro, an effect which could be inhibited by bicalutamide [99]. Separately, it was reported that IGF-1-mediated AR activation required expression of β(1A) integrins, which facilitated functional interaction between the activated IGF-1 receptor and AR, subsequently leading to AR-mediated anchorage-independent growth of PC [100]. Interleukin-6 (IL-6) was shown to activate AR reporter gene constructs in DU145 PC cells and upregulate PSA secretion in LNCaP cells [101]. AR activation by IL-6 has been proposed to be mediated by both the Stat3 and MAPK signaling pathways in PC cells [102, 103]. Specifically, in the absence of androgens, IL-6 was shown to induce association of Stat3 and AR, followed by increased expression of AR-regulated genes [102].

Promotion of AR signaling in PC may also occur through loss of negative regulatory signals, which suppress AR function. This concept is supported by findings demonstrating that the cyclin D1b isoform of the widely studied cell cycle regulator does not possess AR inhibitory function attributed to the more common cyclin D1a isoform [104], and that cyclin D1b was found to be elevated in clinical PC specimens [105]. Additionally, a recent paper demonstrated that cyclin D1b cooperates with AR signaling to promote tumor cell growth and metastatic behavior of PC cells, partially by inducing expression of the pro-oncogenic transcription factor SNAI2 (Slug) [106]. In another example, loss or inactivation of the retinoblastoma (Rb) tumor suppressor gene induced an E2F1-mediated increase in AR mRNA and protein levels, which promoted castrate-resistant growth and resistance to bicalutamide in PC cells [107].

Modulation of AR activity by receptor tyrosine kinases may contribute to progression of PC to castrate-resistant cancer. Overexpression of Her2/neu (ErbB2) has been shown to enhance AR transcriptional activity in both the presence and absence of androgens, stimulating proliferation of LNCaP cells under both settings [108, 109]. Moreover, Src kinase promoted AR transcriptional activity in castrate-resistant C4-2 cells [110]. In line with these findings, several other studies demonstrated that AR transcriptional activity was attenuated by protein kinase inhibitors [101, 111].

## AR-INDEPENDENT MOLECULAR MECHANISMS DRIVING CRPC

### AR-independent bypass pathways

While most research to date has focused on the continued importance of AR in CRPC, alternative signaling pathways supporting proliferation and survival of CRPC cells have been shown to be capable of completely bypassing AR [112–114]. Consequently, AR bypass pathways are not dependent on AR for their downstream effects and can theoretically remain active even in the absence of AR expression [112–114]. Sustained blockade of AR signaling may contribute to the selection of PC cell clones able to upregulate AR bypass pathways, conferring a castrate-resistant phenotype.

Several studies have investigated candidate genes which may support CRPC growth independently of AR. Whitworth and colleagues used an RNA interference (RNAi) phenotypic screen to profile 673 kinases and identify those which contributed most to castrate-resistant growth of LNCaP cells under androgen-depleted conditions [112]. Moreover, expression of the anti-apoptotic protein Bcl-2 was reported to be induced in PC xenograft tumors initially negative for Bcl-2 expression following castration of mice [115]. At the same time, inhibition of Bcl-2 using antisense oligonucleotides delayed onset of castrate resistance in an LNCaP xenograft tumor model [116]. Bcl-2 has been reported to be overexpressed in both mouse models of CRPC as well as clinical samples from CRPC patients [117, 118], although increased Bcl-2 expression is not strictly a prerequisite for progression to CRPC [118, 119].

A recent body of work supports the concept that the glucocorticoid receptor (GR) may be able to substitute for AR in binding to androgen response elements (AREs) and driving PC cell survival under certain circumstances [113, 120, 121]. Chromatin immunoprecipitation followed by sequencing (ChIP-seq) and mRNA expression analysis indicated that these two steroid hormone receptors have highly overlapping cistrome and transcriptome profiles in PC [113, 120]. Furthermore, acquired resistance to enzalutamide in LNCaP cells and xenograft tumors correlated with upregulation of GR expression and was demonstrated to be dependent on GR for enzalutamide-resistant growth [113]. Glucocorticoid-induced activation of GR in VCaP cells was also found to confer resistance to enzalutamide [113]. In the same study, PC xenograft tumors which developed resistance to the second-generation antiandrogens enzalutamide and apalutamide showed a 27-fold higher expression of GR mRNA levels compared to controls [113]. Although research is ongoing, evidence thus far suggests that proliferation and survival signals may be able to bypass AR through GR in PC cells treated with antiandrogens, including second-generation agents. Whether other steroid hormone receptors, such as progesterone receptor (PR), contribute to AR bypass signaling remains to be determined.

### AR-negative cell populations: prostate cancer stem-like cells

The cancer stem cell theory states that only a rare subpopulation of tumor cells possessing stem cell-like properties, most notably unlimited self-renewal capacity, and multilineage differentiation, can initiate tumor formation [122]. The more general term “tumor-initiating cells (TICs)” is also used interchangeably [123] with cancer stem-like cells, and refers to a population of cells within the original clinical cancer sample that has the ability and is critical for forming a tumor when xenotransplanted to immunodeficient mice [124]. In other words, the presence of TICs in patient-derived cancer samples and supportive growth environment for their proliferation in vivo determines the success rates of the tumor formation in mice [124]. Cancer stem cells are believed to be resistant to most therapies used to debulk tumors, harboring the ability to repopulate a tumor with therapy-resistant progeny more prone to metastasis (reviewed in [125, 126]). The gold standard for assessment of putative cancer stem-like cell populations is the capability of the cancer stem-like cells to generate serially transplantable xenograft tumors in immunodeficient mice that histopathologically resemble the parental tumor. Other surrogate assays/markers of cancer stem-like properties include cell surface markers, transcriptional profiles and tumor sphere-forming ability (reviewed in [127]).

Several putative populations of PC stem-like cells (PCSCs) have been reported in the literature, most commonly identified through fluorescence-activated cell sorting (FACS) of bulk tumor cell lines, xenograft tumors or clinical samples for stem-like cell surface markers. Reported marker profiles include CD44+/α2β1+/CD133+ [128], TRA-1-60/CD151/CD166 [129], ALDH [130] and PSA−/lo [131], among others. While PC stem-like cells are positive for CD44 marker, the expression of the standard isoform of CD44 is typically lost in PC due to aberrant splicing[132]. Importantly, a number of reports have suggested that PCSCs are AR-negative or express very low levels of AR, predicting lack of responsiveness to antiandrogens and other therapies targeting the AR signaling axis. Patrawala and colleagues demonstrated that CD44+ PCSCs were negative for AR expression, as well as approximately 10-100x more tumorigenic in immunodeficient mice than CD44- non-PCSCs [133]. Moreover, putative CD44+/α2β1+/CD133+ PCSCs isolated from clinical PCs following prostatectomy were found to be AR-negative and exhibited a prostate basal cell phenotype [128]. More recent work supports enrichment of a basal cell gene expression profile in metastatic CRPC, establishing a link between expansion of a stem-like cell population and castrate resistance [134]. Collectively, this body of work suggests that residual AR-negative/low PCSCs may remain after androgen deprivation, potentially repopulating tumors with castrate-resistant tumor cells.

### AR-negative cell populations: neuroendocrine prostate cancer cells

Interspersed within prostate epithelium are neuroendocrine (NE) cells, which can be identified by morphology under electron microscopy or through positive staining for chromogranin A, synaptophysin, neuron-specific enolase or other markers of NE differentiation [135]. These specialized cells secrete a variety of neuropeptides, such as bombesin, neurotensin, parathyroid hormone-related protein (PTHrP), serotonin and calcitonin, which are known to promote proliferation and survival of prostate adenocarcinoma cells [135]. In the normal prostate, NE cells constitute <1% of the total cell population and typically remain quiescent [135].

Neuroendocrine prostate cancer (NEPC) describes a heterogeneous group of tumors, including prostate adenocarcinoma with NE differentiation, adenocarcinoma with Paneth cell NE differentiation, carcinoid tumors, pure small cell carcinomas, pure large cell NE cancer and adenocarcinomas with mixed histologies [136]. Approximately 5-10% of primary prostate adenocarcinomas contain NE tumor cells, with greatest abundance in high-grade PCs [135, 137]. NEPC can be classified as de novo or treatment-related. NEPC arising de novo is a rare entity, typically constituting less than 2% of all primary PCs and displays a high propensity for metastasis, limited treatment options and dismal prognosis, with most patients succumbing to the cancer within a year of diagnosis [138, 139]. Much more commonly, NEPC emerges in CR recurrent tumors in patients who have previously had androgen deprivation therapy for prostate adenocarcinoma (reviewed in [140–154]), suggesting that epithelial cells are able to transdifferentiate into NE cells and/or NE cells possess a proliferative advantage under androgen deprivation.

NEPC cells are negative for AR and PSA expression and highly proliferative [155]. Recent work by Beltran and colleagues [156] indicates divergent evolution of NEPC cells from one or more CRPC cells rather than linear clonal evolution. Moreover, NE differentiation in PC has been shown to be a predictive factor of unfavorable clinical outcome [147, 157–160]. Most importantly, emergence of NE differentiation during PC progression to CRPC has therapeutic implications. Specifically, molecular mechanisms driving AR-independent/negative NE cells in CRPC need to be identified for development of strategies to therapeutically target NE cell populations in CRPC.

The lack of AR expression in NEPC cells [135, 137] has led to efforts to determine the key signaling pathways that are dysregulated in these cells. Molecular profiling of NEPC revealed that deletion of RB1 and PTEN, mutations in TP53 and amplification of MYCN and Aurora kinase A (AURKA) are common in NE cells [161, 162]. Work from the Huang group demonstrated that mutant p53 inactivates the IL-8/CXCR2/p53 signaling pathway which maintains NE cells in a quiescent state [163] and simultaneously results in AURKA overexpression, leading to hyperproliferation of NE cells [164]. Overexpression of N-Myc in LNCaP cells induced NE differentiation, downregulated AR levels and decreased AR target gene expression [162]. Moreover, the Witte group showed that aberrant N-Myc expression combined with activated AKT1 was sufficient to transform human prostate epithelial cells into NEPC with phenotypic features characteristic of metastatic CRPC in patients [165]. With the growing clinical use of abiraterone and enzalutamide to suppress AR signaling, NE differentiation may become an increasingly common resistance mechanism underlying castrate resistance.

## JAK2-STAT5 SIGNALING PROMOTES GROWTH OF PROSTATE CANCER THROUGH AR-DEPENDENT AND AR-INDEPENDENT MOLECULAR MECHANISMS

### Jak2-Stat5a/b signaling pathway in prostate cancer

In order to target AR-negative cell populations in CRPC and to impose an additional survival strain for AR-positive PC cells during ADT, identification of non-AR therapeutic targets may provide novel treatment strategies for CRPC. Stat5a/b are latent cytoplasmic proteins that function as both signaling molecules and nuclear transcription factors, which are critical for PC cell viability in vitro, in vivo and in patient-derived PCs ex vivo [166–174]. Two highly homologous isoforms, Stat5a and Stat5b (hereafter referred to as Stat5a/b), display over 90% amino acid identity and are encoded by genes juxtaposed on chromosome 17q21.2 [175].

In PC, Stat5a/b is predominantly activated by binding of prolactin (Prl) to the membrane-bound prolactin receptor (PrlR), which transmits signals through Jak2 kinase [173, 176, 177]. In addition, Stat5a/b may also be activated by Src kinase or members of the EGF receptor family [178–180]. Prl is a polypeptide hormone whose actions are mediated by the PrlR, a non-kinase single-pass transmembrane receptor part of the class 1 hematopoietic cytokine receptor superfamily (reviewed in [181, 182]). Prl is primarily secreted by lactotroph cells of the anterior pituitary gland and carries out a wide variety of functions, including the well-known effect of stimulating lactation in females; circulating Prl is present in males, though at lower levels than in females (reviewed in [181, 182]). A large body of work provides evidence that Prl is expressed within the prostate epithelium and functions as a local growth factor in an autocrine/paracrine manner distinct from its endocrine mechanism [183–187]; (reviewed in [188]).

**Figure 2:**
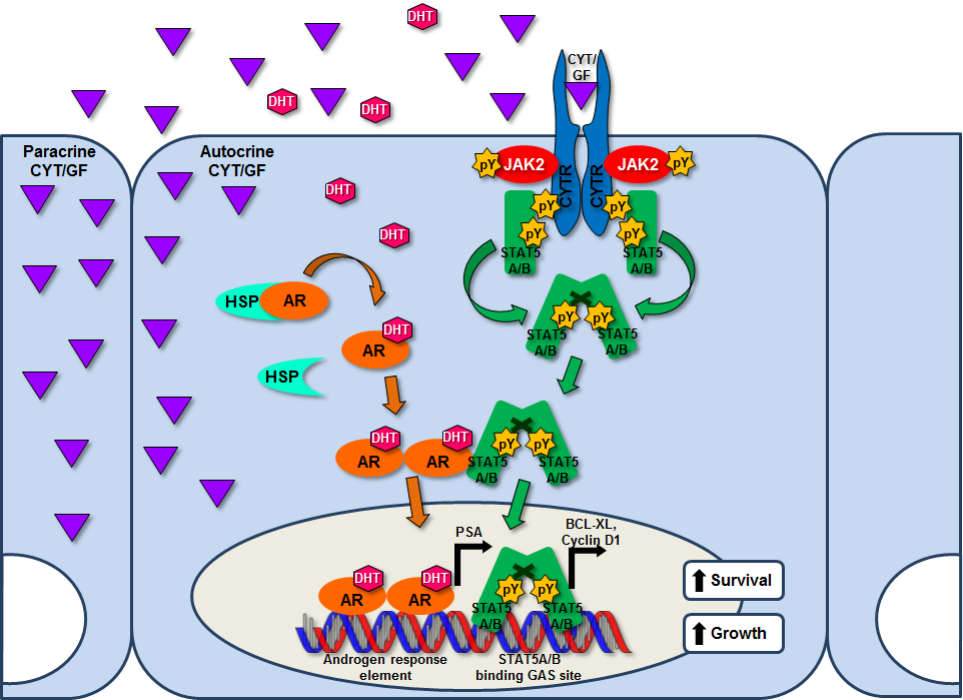
Canonical Jak2-Stat5a/b signaling in prostate cancer cells and functional interaction with AR signaling Various levels of inhibition are possible in the Stat5a/b signaling cascade to block Stat5a/b target gene expression, including targeting of cytokine receptor activation, Jak2 kinase-mediated phosphorylation, and Stat5a/b dimerization, DNA binding and transcriptional activity. Physical interaction between Stat5a/b and AR has been reported, providing a mechanistic basis for crosstalk between these two pathways, which results in reciprocal synergistic effects on nuclear localization and transcriptional activity. Depicted are known Stat5a/b target genes Bcl-xL and cyclin D1 and AR target gene PSA. Abbreviations: AR, androgen receptor; ARE, androgen response element; CYT, cytokine; CYTR, cytokine receptor; DHT, dihydrotestosterone; GAS, gamma-interferon activation sequence; GF, growth factor; HSP, heat shock protein; pY, phosphotyrosine.

Signaling through the Jak2-Stat5a/b pathway is initiated upon Prl binding to PrlR and subsequent receptor-associated Jak2 kinase activation by autophosphorylation. This leads to phosphorylation of cytoplasmic tyrosine residues of the cytokine receptor which serves as docking sites for Stat5a/b via binding of the Stat5a/b SH2 domain [189]. Stat5a/b monomers recruited to the receptor-tyrosine kinase complex are activated by tyrosine kinase-mediated phosphorylation at conserved C-terminal tyrosine residues (Stat5a: Y694, Stat5b: Y699) (reviewed in [190]). Phosphorylated Stat5a/b (pY694/699) monomers undergo homo- or heterodimerization, followed by nuclear localization of functional Stat5a/b dimers in an active, energy-dependent process directed by Ran-dependent nuclear import machinery [191, 192]. Specifically, a nuclear shuttling complex is formed by the chaperone protein MgcRacGAP, the small G protein Rac1 and the carrier protein importin/karyopherin β1, which cooperate to translocate the Stat5a/b dimer from cytosol to nucleus [193]. Within the nucleus, Stat5a/b dimers bind to palindromic DNA consensus sequences (TTC(C/T)N(G/A)GAA), referred to as gamma-interferon activation (GAS) elements, to initiate transcription of Stat5a/b regulated genes [194–196]. Homodimers of Stat5a/b have equivalent specificity for binding palindromes spaced three base pairs apart [196, 197], while tetramers of Stat5a/b can bind tandem consensus and non-consensus GAS motifs spaced six base pairs apart [196]. The sequence of molecular events in the canonical Jak2-Stat5a/b signaling cascade leading to activation of Stat5a/b-regulated genes is shown in Figure 2.

### Stat5a/b is a predictive biomarker and therapeutic target in prostate cancer

Over the last decade, numerous reports have supported the role of Stat5a/b in clinical progression of PC. Stat5a/b is constitutively active in human PC, but not in normal prostate epithelium [174]. Examination of the distribution of active Stat5a/b across 114 paraffin-embedded clinical PC specimens of various histological grades revealed that active Stat5a/b was strongly associated with high-grade PC [173]. In a separate study, analysis of tissue microarrays derived from 357 PC patients accompanied by 30-year clinical follow-up data found that high expression levels of active Stat5a/b were correlated with poorly differentiated, high-grade PC [172]. Importantly, active Stat5a/b status was predictive of early PC recurrence after initial treatment by radical prostatectomy or transurethral resection [172]. Active Stat5a/b was found to be an independent prognostic marker of early cancer recurrence not only in high-grade PC but also in intermediate grade PC [172]. Patients who had received androgen deprivation therapy prior to radical prostatectomy were more likely to display active Stat5a/b when compared to patients not receiving neoadjuvant therapy [172]. More recent work analyzing nuclear Stat5a/b expression levels in two cohorts of PC patients treated by radical prostatectomy or deferred palliative therapy showed that nuclear Stat5a/b was an independent prognostic marker for both cohorts [166]. Corroborating earlier work, high levels of nuclear Stat5a/b predicted increased risk of early cancer recurrence following radical prostatectomy in PCs of intermediate grade [166]. At the same time, active Stat5a/b predicted early PC-specific death in the deferred palliative therapy cohort [166]. In the cohort of PC patients treated by radical prostatectomy, each patient was represented by 10 tissue cores taken from different areas of the tumor. Interestingly, the presence of active Stat5a/b even in one of the 10 tissue cores already predicted likely cancer recurrence after radical prostatectomy [166], suggesting that those PCs with elevated nuclear Stat5a/b anywhere in the tumor may already have micrometastasized at the time of surgery. Further work demonstrated that the Stat5a/b gene locus is amplified in approximately 30% of CRPC metastases [198]. Similar to the presence of nuclear Stat5a/b protein in specific areas of prostate tumor, Stat5a/b locus amplification was dispersed into groups of PC cells within the tumor tissue, as demonstrated by FISH of paraffin-embedded tissue sections. It is important to note, however, that presence of specific areas with Stat5a/b gene locus amplification shown by FISH may not be detectable in analyses of RNA/DNA extracted from ground whole tissue specimens which include a mix of both epithelial and stromal components. Collectively, these findings indicate that Stat5a/b may provide a robust biomarker for recurrence of lethal PC after failure of first-line therapy for early stage cancer, warranting further investigation.

### Stat5a/b promotes growth of prostate cancer cells and xenograft tumors

A substantial body of work supports the concept that Stat5a/b is highly critical for the viability of human PC cells in vitro and xenograft tumor growth in vivo. Inhibition of Stat5a/b by varied methods induced massive apoptotic death of PC cells in culture and suppressed the growth of xenograft tumors in nude mice [170, 171, 174, 199]. Ahonen and colleagues provided the first evidence of Stat5a/b in promotion of PC growth [174]. Transduction of PC cells with adenovirus expressing dominant-negative Stat5a/b lacking the transactivation domain, induced extensive apoptosis of PC cells, as determined by cell morphology, DNA fragmentation, caspase-3 and -9 activation, and cell viability assays [174]. Stat5a/b regulation of PC cell viability was further validated in the transgenic adenocarcinoma mouse prostate (TRAMP) model. In TRAMP-derived PC cell lines, use of an inducible carboxy terminal-truncated Stat5a/b construct to inhibit wild-type Stat5a/b activity led to decreased PC cell growth in soft agar and tumor formation in nude mice [200]. More recent studies further indicated a critical role of Stat5a/b in maintenance of PC viability, as inhibition of Stat5a/b by various methodological approaches (antisense oligonucleotides, RNA interference or adenoviral expression of dominant-negative Stat5a/b) triggered extensive apoptosis across multiple Stat5a/b-positive PC cell lines. Mechanistically, Stat5a/b was found to sustain viability and promote cell cycle progression of PC cells through activation of Bcl-xL and cyclin D1, respectively [171]. Based on gene array analyses, target genes regulated by Stat5a/b include genes regulating proliferation, apoptosis and metastatic processes [169, 170]. These results also translated to the in vivo setting, with inhibition of Stat5a/b decreasing the incidence and growth of both subcutaneous and orthotopic PC xenograft tumors in nude mice [171]. Most importantly, inhibition of Stat5a/b blocked not only primary growth of PC xenograft tumors, but also recurrent growth of castrate-resistant tumors [177] demonstrated by pharmacological inhibition of Stat5a/b signaling by a potent Jak2 kinase inhibitor, AZD1480, in the CWR22Pc model of CRPC growth. In fact, Stat5a/b inhibition was more efficacious than docetaxel chemotherapy at both preventing recurrence of castrate-resistant CWR22Pc tumors following androgen deprivation as well as suppressing growth of established castrate-resistant CWR22Pc tumors [177].

### Stat5a/b induces metastatic behavior and stem-like cell properties of prostate cancer cells

The first evidence of Stat5a/b promotion of metastatic behavior of PC cells in vitro and in vivo [169] was provided using an experimental lung metastasis model; inoculation of nude mice with PC cells expressing an active Stat5 construct (Stat5aS710F) increased in vivo formation of lung metastases by 11-fold compared to β-galactosidase (LacZ)-expressing control cells [169]. Active Stat5a/b upregulated hallmarks of the epithelial-to-mesenchymal transition (EMT) that precedes metastasis, including enhanced migration, decreased expression of cell surface E-cadherin, increased expression of mesenchymal markers and heterotypic adhesion of both AR-positive and AR-negative PC cells to endothelial cells [169, 201]. In conjunction with promotion of EMT, Stat5a/b induced stem-like properties in PC cells [201], such as tumor sphere formation and expression of the cancer stem cell marker Bmi1, a core component of Polycomb-Repressive Complex 1 (PRC1), which overrides cellular senescence and promotes self-renewal [202–204]. Mechanistically, Stat5a/b regulation of both EMT and cancer stem-like cell properties were found to be mediated by Twist1, a well-characterized EMT transcription factor [205–207]. Indeed, genetic knockdown of Twist1 suppressed Stat5a/b-induced functional endpoints of EMT, and Stat5a/b-induction of stem-like features in PC cells. These findings establish Stat5a/b in the promotion of EMT processes and a cancer stem-like cell phenotype, which enable the formation of distant PC metastases. Pharmacological targeting of Stat5a/b may prevent metastatic dissemination of PC.

### Stat5a/b induces AR activity in prostate cancer cells, but also promotes PC cell growth independently of AR

Stat5a/b has been shown to promote AR signaling in PC cells (Figure 2). The first paper to report functional interaction between Stat5a/b and AR demonstrated that active Stat5a/b increased nuclear localization and transcriptional activity of AR in PC cells [208]. Moreover, Stat5a/b and AR were reported to physically interact in PC cells [208]. Follow-up studies implicated Stat5a/b in regulating proteasomal degradation of androgen-liganded AR in PC cells [209]. Specifically, Stat5a/b was shown to physically interact with and sequester AR liganded by antiandrogens, including enzalutamide, from trafficking to the proteasome [209]. Furthermore, antiandrogen therapy induced proteasomal degradation of AR in PC cells, a degradative mechanism which can be accelerated by disruption of Stat5a/b expression or activation [209]. A high-profile study using ChIP-Seq to map genome-wide occupancy of AR in clinical CRPC specimens revealed that AR binding sites in clinical PCs differed markedly from those in human PC cell lines. Importantly, novel AR binding sites identified in CRPC patients were strongly enriched for Stat, Myc and E2F binding motifs, suggesting that genomic repositioning of AR in CRPC may be induced by cooperating transcription factors in clinical CRPC [210]. Notably, this work also demonstrated that physical interaction of Stat5a/b and AR occurs in clinical CRPC specimens [210].

Importantly, Stat5a/b blockade, by either genetic or pharmacological knockdown, induces extensive apoptotic death of PC cells that do not express AR. Specifically, inhibition of Stat5a/b was found to induce rapid apoptotic death of DU145 PC cells, which are negative for AR expression, providing evidence that Stat5a/b sustains viability of PC cells independently of AR [170, 211]. At the same time, Stat5a/b overexpression in DU145 cells increased colony formation in vitro and accelerated xenograft tumor growth in vivo [198]. These findings indicate that Stat5a/b acts through mechanisms operating independently of AR to promote growth of PC, in addition to mechanisms directly impacting AR function. Future challenges include identification of the specific mechanisms underlying the capability of Stat5a/b to maintain and promote viability of PC cells independently of AR. Moreover, it will be important to determine if Stat5 inhibition will be able to induce death of AR-negative cell populations in castrate-resistant prostate tumors.

### Pharmacological targeting of Stat5a/b as a treatment strategy for prostate cancer

Recent studies demonstrate that enzalutamide provides a modest improvement in survival in patients with PC due to rapid development of resistance [8, 9]. Moreover, in a preclinical model, enzalutamide-resistant xenograft tumors treated with an experimental third-generation antiandrogen responded for only eight weeks before resistance developed again [212]. A treatment strategy that targets both AR and Stat5a/b concurrently potentially provides an additive or even synthetic lethal approach to kill PC cells as well as decrease the likelihood of selection for resistance mechanisms to the new-generation anti-androgens.

Pharmacological inhibition of the Stat5a/b signaling pathway can be accomplished through blockade at various levels, with inhibitors directed against the cytokine/growth factor receptor, tyrosine kinase or Stat5a/b itself. Of note, highest specificity with least clinical side-effects is likely achieved through direct targeting of Stat5 instead of its upstream activators. Indeed, past work has shown that disruption of Stat5a/b dimerization, transactivation and/or DNA binding is possible by targeting of the appropriate domain(s). Initial approaches utilized a Stat5a/b dominant-negative mutant lacking the transactivation domain [171, 174] and a Stat3 decoy phosphotyrosyl peptide [213, 214] to demonstrate proof-of-principle of direct Stat transcription factor inhibition. More recent investigations have shown that natural or synthetic small molecule compounds are capable of inhibiting Stat5a/b activity and downstream signaling (Table 1).

**Table 1:** Comparison of selected Stat5a/b inhibitors in efficacy of inhibition of Stat5a/b tyrosine phosphorylation (pYStat5a/b), as detected by Western blot analysis

**Publication (Fig. of Interest):**	**Inhibitor Name:**	**pYStat5a/b Inhibition (µM):**
Nam et al. 2012 (Fig. 2A)	E804	10-20
Nelson et al. 2011 (Fig. 2A)	Pimozide	>10
Elumalai et al. 2015 (Fig. 4C)	Stafib-1 (Comp. #17)	10
Müller et al. 2008 (Fig. 3A)	Comp. #6	400
Page et al. 2012 (Fig. 2A)	BP-1-107 BP-1-108 SF-1-087 SF-1-088	40
Cumaraswamy et al. 2014 (Fig. 4A)	AC-3-019	15
Liao et al. 2015 (Fig. 2F)	IST5-002	5

**Figure 3:**
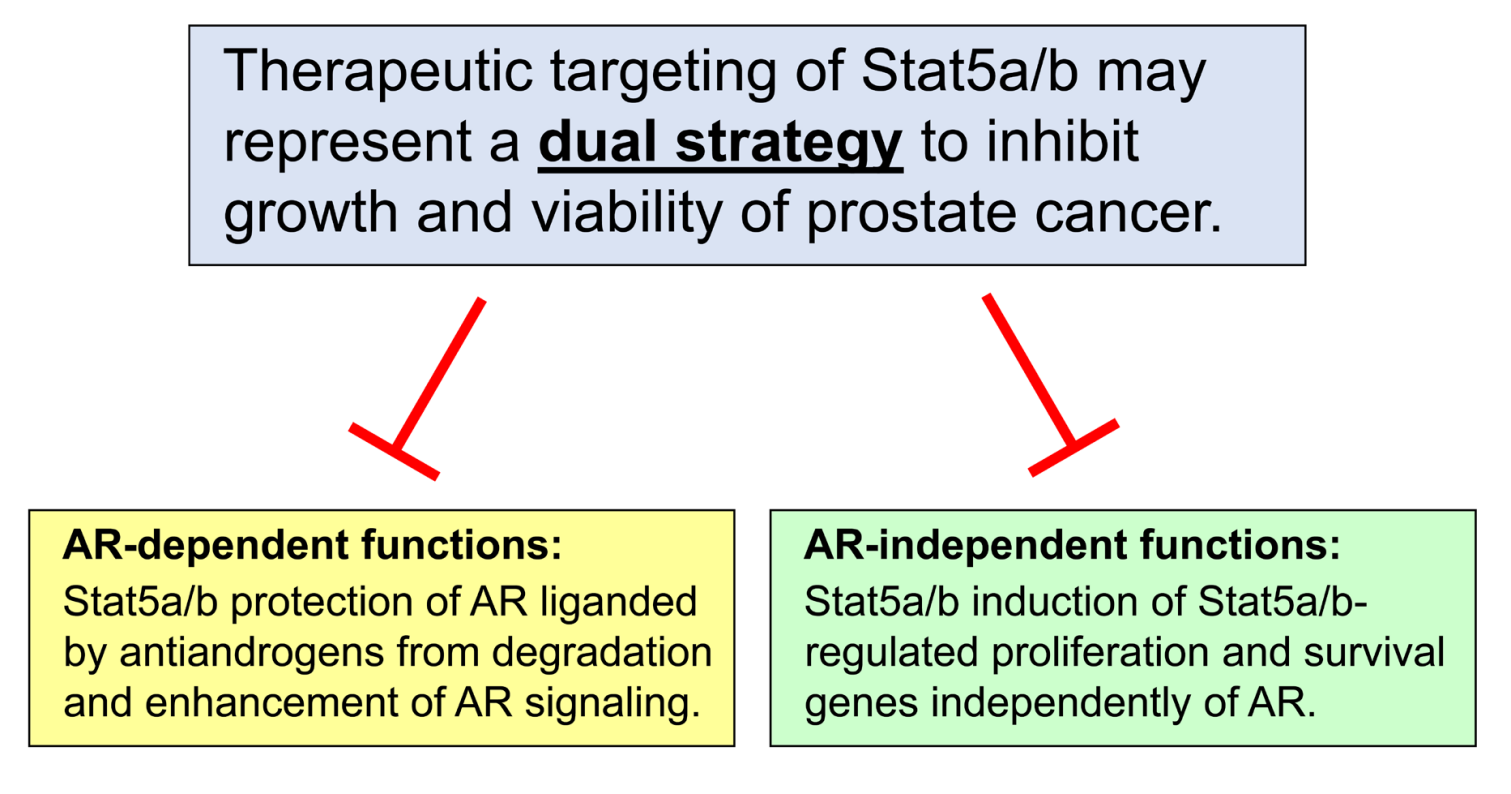
Therapeutic targeting of Stat5a/b inhibits growth of prostate cancer through AR-dependent and AR-independent mechanisms Disruption of Stat5a/b signaling inhibits growth and viability of prostate cancer through two distinct mechanisms, serving as a potential dual strategy to eliminate prostate cancer cells. First, targeting of Stat5a/b inhibits the abilities of Stat5a/b to protect antiandrogen-liganded AR from proteasomal degradation and enhance AR signaling, including downstream molecular events such as AR nuclear localization, chromatin binding, and activation of target gene expression in prostate cancer cells. Second, targeting of Stat5a/b prevents the induction of AR-independent, Stat5a/b-regulated genes involved in proliferation and survival of prostate cancer cells.

Compounds which are currently in clinical use for a variety of oncologic and non-oncologic indications have been found to inhibit Stat5a/b in several preclinical studies (Table 1). Nam and colleagues screened a number of indirubicin derivatives, demonstrating that the most potent compound (E804) blocked Stat5a/b phosphorylation at 10-20 µmol/L in K562 human chronic myelogenous leukemia (CML) cells positive for Bcr-Abl, which drives constitutive activation of Stat5a/b [215]. Pimozide (Orap®), an antipsychotic drug, was shown to inhibit Stat5 phosphorylation in K562 cells by 35-55% at 10 µmol/L [216]. Other investigators have pursued rational drug design strategies in development of Stat5a/b inhibitors (Table 1). Elumalai and colleagues used fosfosal, a salicylic acid derivative and previously identified inhibitor of Stat5b [217], as a lead compound for optimization studies targeting the Stat5b SH2 domain [218]. The most promising compound, known as #17 or Stafib-1 (Stat five b inhibitor-1), specifically inhibited Stat5b phosphorylation in K562 cells at an IC50 of approximately 10 μmol/L [218]. In another study, a lead compound identified from a 17,000 molecule library was modified with chemical substitutions to yield a family of Stat5a/b small molecule inhibitors, of which compound #6 blocked IFN-α-stimulated Stat5a/b phosphorylation in human Burkitt's lymphoma B cells at approximately 400 µmol/L [219]. A different investigator group generated a series of SH2-domain binding, salicylic acid-derived Stat5a/b inhibitors (BP-1-107, BP-1-108, SF-1-087, SF-1-088), which were all capable of fully blocking Stat5a/b phosphorylation at 40 µmol/L [220]. Building on this work, the investigators used one of their previously identified Stat5a/b inhibitors as a scaffold for further optimization [221]. A new library of 24 compounds was generated, of which AC-3-019 emerged as the most promising candidate, inhibiting Stat5a/b phosphorylation at 15 µmol/L in K562 cells [221].

Recently, the Nevalainen group identified and validated a small molecule inhibitor which targets the Stat5a/b SH2 domain in both PC and CML, malignancies known to be driven by Stat5a/b signaling [211]. In silico screening of chemical structure databases and medicinal chemistry modeling yielded 30 top-ranked candidate molecules, of which the lead compound IST5-002 proved most potent in biological assays [211]. Specifically, IST5-002 blocked transient docking of the SH2-domain of the Stat5 monomer to the phosphotyrosyl moiety of a tyrosine kinase-receptor complex, resulting in inhibition of both Jak2 and Bcr-Abl-mediated phosphorylation of Stat5. Secondly, dimerization of Stat5 was inhibited by IST5-002, which may be caused by decreased Stat5 phosphorylation or additional suppression of binding of the phosphorylated Stat5 monomer to the pY694/699 residues of the partner Stat5 monomer. Nuclear translocation, DNA binding and transcriptional activity of Stat5 were all inhibited at considerably low IC50 values, while IL-6-stimulated transcriptional activation of Stat3 was unaffected [211]. IST5-002 triggered extensive apoptosis of PC cells in culture, suppressed growth of PC xenograft tumors in nude mice and induced death of patient-derived PCs in ex vivo organ cultures [211]. IST5-002 also triggered apoptosis of both imatinib-sensitive and imatinib-resistant CML cell lines and induced death of primary CML cells [211]. Importantly, IST5-002 inhibited Bcr-Abl-driven Stat5a/b phosphorylation in K562 cells at an IC50 of less than 5 µM and, thus, IST5-002 is more potent in cell-based assays than any existing published Stat5a/b inhibitors (Table 1). Moreover, IST5-002 is the first small molecule inhibitor of Stat5a/b to demonstrate efficacy in experimental models of solid and hematological malignancies at low micromolar potency, providing a highly attractive lead structure for further optimization and clinical development.

## CONCLUSIONS

PC remains a leading cause of cancer-related death for American men, with a subset of patients progressing to metastatic CRPC despite receiving aggressive treatment for localized cancer. An improved understanding of the molecular biology driving cancer progression has allowed substantial expansion of the therapeutic landscape for CRPC. Over the past five years, a diverse array of novel agents has been approved by the FDA for use in CRPC, with many other experimental therapies now in late-stage clinical trials. Most notable in the newly expanded treatment options for CRPC are the androgen synthesis inhibitor abiraterone and second-generation antiandrogen enzalutamide, both of which have been widely adopted in clinical practice by medical oncologists. However, additional studies are required to optimally integrate these new and emerging therapies with existing standard-of-care treatment paradigms. In addition, drug sequencing strategies need to be designed to anticipate and overcome newly described resistance mechanisms. Despite the impressive progress to date, the lack of a curative therapy for CRPC underscores a remaining unmet need, which stems from the still incompletely understood transition from androgen-dependence to castrate-resistance.

Identification of therapeutic targets which regulate non-AR-mediated proliferation and survival pathways represent an opportunity to improve treatment for advanced PC. Stat5a/b has been previously validated as a biomarker and therapeutic target in PC. Active Stat5a/b expression levels carry prognostic value by identifying patients at risk for cancer recurrence and poor clinical outcomes. Levels of active Stat5a/b carry predictive value as well, serving to predict treatment response to radical prostatectomy in patients with early stage PC. There is a strong rationale for pharmacological targeting of Stat5a/b based on its pleiotropic effects on sustaining PC growth and viability, enhancement of AR transcriptional activity and promotion of metastatic behavior. Stat5a/b serves not only as a critical survival factor for AR-positive but also AR-negative PC cells and xenograft tumors, providing a means for targeting PC outside of the AR signaling axis. The collective findings described in this review indicate that Stat5a/b acts through both AR-dependent and AR-independent molecular mechanisms, which are distinct but not mutually exclusive and therefore hold the potential for imposing synthetic lethal effects if blocked simultaneously. Pharmacological inhibition of Stat5a/b may thus represent a dual strategy to target growth of PC and delay onset of resistance to agents targeting AR in PC.
